# Radiating Amyloid Fibril Formation on the Surface of Lipid Membranes through Unit-Assembly of Oligomeric Species of α-Synuclein

**DOI:** 10.1371/journal.pone.0047580

**Published:** 2012-10-15

**Authors:** Jung-Ho Lee, Chul-Suk Hong, Soonkoo Lee, Jee-Eun Yang, Yong Il Park, Daekyun Lee, Taeghwan Hyeon, Seunho Jung, Seung R. Paik

**Affiliations:** 1 School of Chemical and Biological Engineering, Institute of Chemical Processes, College of Engineering, Seoul National University, Seoul, Korea; 2 Department of Bioscience and Biotechnology, Konkuk University, Seoul, Korea; UCL Institute of Neurology, United Kingdom

## Abstract

**Background:**

Lewy body in the substantia nigra is a cardinal pathological feature of Parkinson's disease. Despite enormous efforts, the cause-and-effect relationship between Lewy body formation and the disorder is yet to be explicitly unveiled.

**Methodology/Principal Findings:**

Here, we showed that radiating amyloid fibrils (RAFs) were instantly developed on the surface of synthetic lipid membranes from the β-sheet free oligomeric species of α-synuclein through a unit-assembly process. The burgeoning RAFs were successfully matured by feeding them with additional oligomers, which led to concomitant dramatic shrinkage and disintegration of the membranes by pulling off lipid molecules to the extending fibrils. Mitochondria and lysosomes were demonstrated to be disrupted by the oligomeric α-synuclein via membrane-dependent fibril formation.

**Conclusion:**

The physical structure formation of amyloid fibrils, therefore, could be considered as detrimental to the cells by affecting membrane integrity of the intracellular organelles, which might be a molecular cause for the neuronal degeneration observed in Parkinson's disease.

## Introduction

Amyloidogenesis, insoluble protein nanofibril formation from innocuous soluble proteins, is the common phenomenon found in various neurodegenerative disorders such as Alzheimer's, Parkinson's, and Prion diseases although actual mechanisms of the cell death in relation to amyloidogenesis remain largely unknown [1]–[3]. Instead of the prevailing pursuit of chemical and biological causes for cytotoxicity, physical and mechanical effects of amyloidogenesis directly responsible for the cellular degeneration have been investigated in this study to unveil molecular etiology of the neurodegenerative disorders.

Despite the complexity of intricate cellular activities, cells are well-organized through functional compartmentalization. Potential toxic substances such as diverse hydrolytic enzymes and biomolecules involved in redox reactions are sequestered within membrane-enclosed organelles such as lysosomes and mitochondria. Their latent toxic activities contained within the membrane structures are then coordinated to achieve normal cellular biogenesis. Alternatively, the membrane-enclosed organelles are also considered as potential toxic source as they become disrupted by the physical structures like amyloid fibrils.

Besides the prevalent nucleation-dependent fibrillation, we have recently proposed a distinctive mechanism of amyloid fibril formation named double-concerted fibrillation with a Parkinson's disease (PD) related amyloidogenic protein α-synuclein. Its instantaneous amyloid fibrillation was demonstrated with the oligomers of α-synuclein in the presence of 0.5% hexane [4]. This oligomeric unit assembly process was also evident in the shear-induced fibrillation, which resulted in the curly amyloid fibrils distinctive from the straight fibrils obtained via nucleation-dependent fibrillation process of monomeric α-synuclein [5]. It was the curly fibrils that formed transparent amyloid hydrogel comprised of fine nanospacing. The oligomeric unit assembly was finally proved with the α-synuclein–coated gold nanoparticles by producing the peapod-type chains of gold nanoparticles encapsulated within dielectric amyloid nanofibrils of α-synuclein which were demonstrated to exert photoconductance with visible light [6]. Taken together, the oligomers of α-synuclein readily assemble into the fibrillar suprastructures as their internal structures become subtly rearranged.

Since α-synuclein forms abundant radiating filaments of the Lewy bodies, a pathological hallmark of PD [7]–[9], in which another major component of lipids is shown to coexist [10], the oligomeric unit assembly of α-synuclein has been examined with lipid membranes. The amphipathic environment of lipid membranes could influence the oligomeric state to elicit the subtle structural rearrangement prerequisite for the concerted amyloid fibril formation on the surface of membrane. Since the presumable suprastructure formation could cause membrane disruption, various toxic substances would be released from intracellular organelles during cellular degeneration. Mechanical disruption of the lipid membranes via the physical suprastructure formation, therefore, could be considered as an alternative cell death mechanism to the general notion of cell death directed by chemical effects of biomolecules.

## Results

### Radiating amyloid fibrils on the surface of lipid membranes

**Figure 1:**
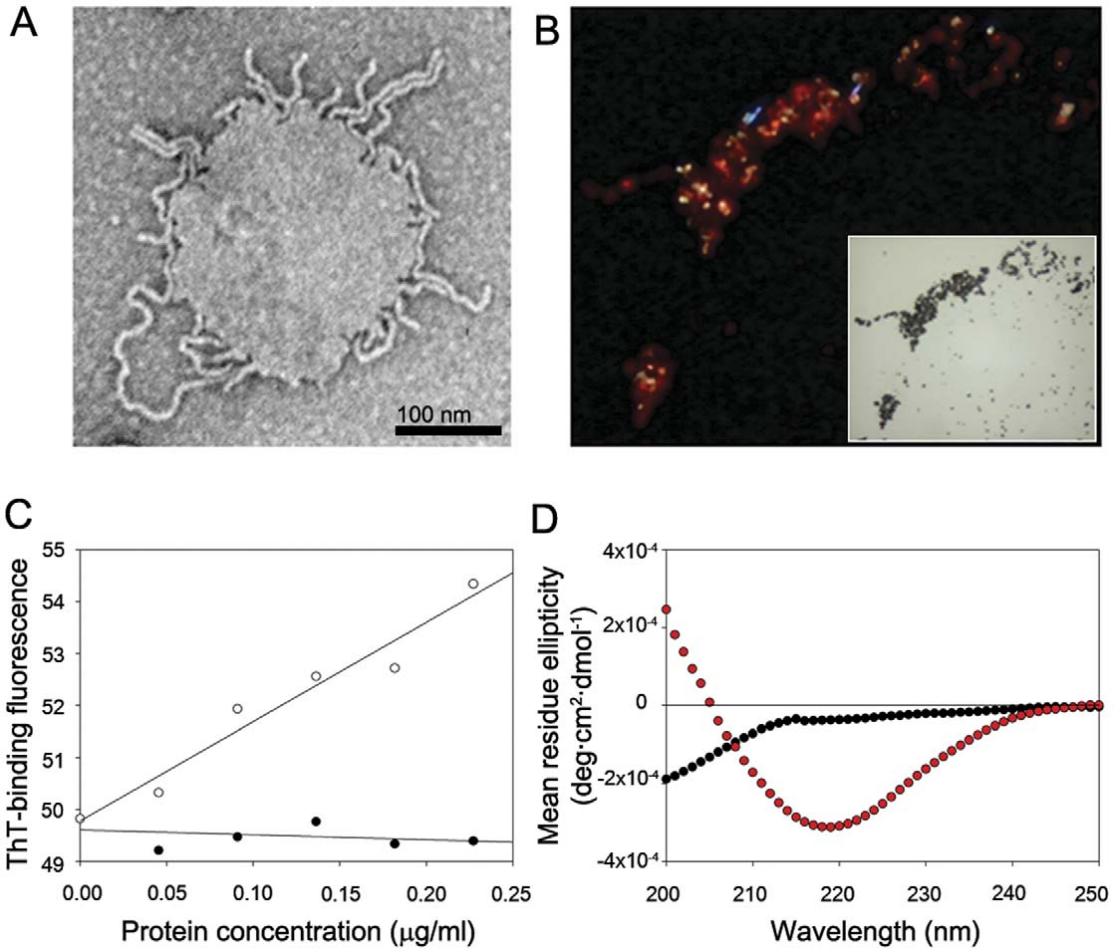
Radiating amyloid fibril (RAF) formation on the surface of lipid membranes. (**A**) Transmission electron microscopy (TEM) image of radiating fibrils that were developed on the surface of liposomes. (**B**) With congo red staining, birefringency of the radiating fibrils on PC-liposomes has been monitored with fluorescence microscope under polarized light. The corresponding image revealed with light microscope of differential interference contrast (DIC) is also shown in the inset. (**C**) Thioflavin-T (ThT) binding fluorescence of the PC-liposomes (0.57 mg/ml) treated with either the oligomers (open dots) or the monomers (closed dots) of α-synuclein at their protein concentrations indicated was monitored at 482 nm with an excitation at 450 nm. (**D**) Circular dichroism (CD) spectra obtained for the PC-liposomes (1.25 mg/ml) incubated with either the oligomers (red dots) or the monomers (black dots) of α-synuclein at 0.5 mg/ml after an extended period of quiescent incubation for 18 hours at 37°C.

Remarkable development of radiating amyloid fibrils (RAFs) from the surface of lipid membranes was observed as the α-synuclein oligomers were incubated with the liposomes made of phosphatidylcholine (PC) for less than 5 min in 20 mM Mes, pH 6.5, under quiescent condition at room temperature (**Figure 1A**). The oligomers were collected as previously described in the middle of lag phase of the aggregation kinetics of α-synuclein monitored with thioflavin-T (ThT) binding fluorescence (**Figure S1**) [4], [5]. Upon congo red staining, the fibril-containing suprastructures exhibited the birefringency under fluorescence microscope (**Figure 1B**). ThT binding fluorescence of the fibril-membrane complex also linearly increased as the amount of oligomers was raised while the ThT binding fluorescence was hardly affected with monomeric α-synuclein (**Figure 1C**). Circular dichroism (CD) spectroscopic analyses of the liposomes treated with either the oligomers or the monomers of α-synuclein following an extensive period of incubation for 18 hr at 37°C indicated that the β-sheet structure was observed only with the oligomer-treated membranes by showing minimum molar ellipticity at 218 nm whereas the monomer-treated liposomes failed to show the β-sheet conformation but retaining the original random structure of α-synuclein with minimum ellipticity at 200 nm (**Figure 1D**, and **Figure S2**). Taken together, the radiating fibrils on the membrane surface were demonstrated to be the β-sheet-containing regular nanostructures of amyloid fibrils prone to accommodate the dyes of congo red and ThT.

### β-Sheet free oligomers responsible for the RAF formation

**Figure 2:**
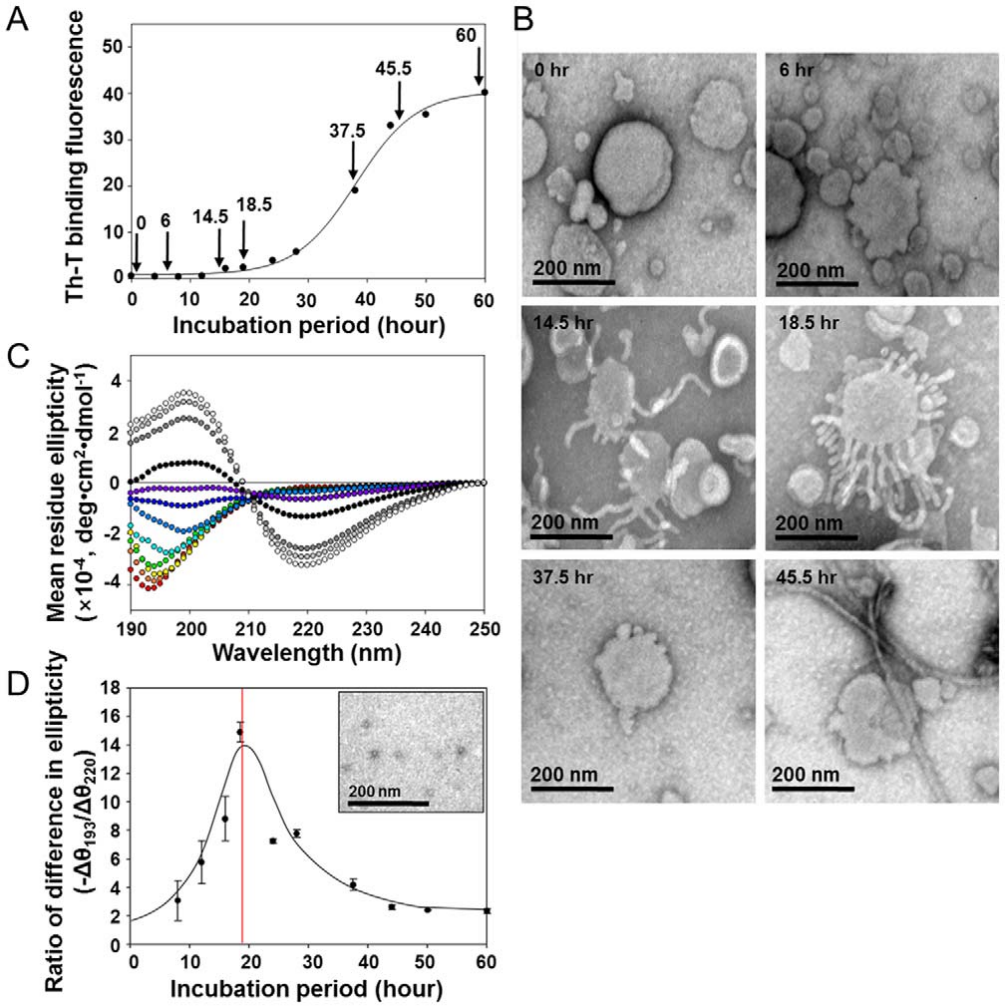
Characterization of the RAF-forming oligomeric species of α-synuclein. (**A**) The fibrillation kinetics of α-synuclein was monitored with ThT binding fluorescence following the incubation of α-synuclein at 1 mg/ml in 20 mM Mes, pH 6.5, at 37°C with shaking at 200 rpm. (**B**) TEM images showing the RAF formation on the surface of PC-liposomes. The liposomes (1.25 mg/ml) were treated with various states of α-synuclein (0.5 mg/ml) collected during the fibrillation kinetics at the times indicated with arrow heads in panel (A). (**C**) CD spectra of the α-synuclein collected at various time points during the fibrillation process (0 hr, red; 4 hr, orange; 8 hr, yellow; 12 hr, green; 16 hr, cyan; 18.5 hr, light blue; 24 hr, blue; 28 hr, violet; 37.5 hr, black; 44 hr, dark gray; 50 hr, gray; 60 hr, white color). (**D**) Plot of negative ratios of the differences in molar ellipticities obtained at 193 nm and 220 nm (-Δθ_193 nm_/Δθ_220 nm_) for the various states of α-synuclein in comparison with those of the monomeric form. The inset shows the oligomeric state of α-synuclein visualized with TEM giving rise to the maximum value of - Δθ_193 nm_/Δθ_220 nm_.

The radiating amyloid fibril (RAF) formation on the surface of lipid membranes was highly dependent upon the oligomeric state of α-synuclein. The RAF formation was performed by incubating PC-liposomes with various states of α-synuclein from monomers and oligomers to amyloid fibrils isolated during the aggregation kinetics (**Figure 2A**). The fibrillation was carried out *in vitro* with α-synuclein in 20 mM Mes, pH 6.5, at 37°C under shaking incubation at 200 rpm, and monitored with ThT binding fluorescence. Aliquots collected at several time points were combined with the membranes, incubated for 5 min at room temperature, and examined with transmission electron microscope (TEM). Intriguingly, discrete RAF formation was observed predominantly with the α-synuclein oligomers obtained at 18.5 hr. The other α-synucleins, however, mostly failed to form RAFs although the species collected at either 14.5 hr or 37.5 hr produced a trace of RAFs on the membranes (**Figure 2B**). The preformed amyloid fibrils obtained at 45.5 hr existed independently from the liposomes (**Figure 2B**, and **Figure S3**). The data suggest that a specific oligomeric state of α-synuclein is crucial to exhibit the unit assembly leading to the RAF formation.

To define the RAF-forming oligomeric state, the α-synucleins collected at various stages were examined with CD spectroscopy and their spectra were analyzed by comparing with the random state of monomeric α-synuclein (**Figure 2C**). As the protein assembled to the final amyloid fibrils, the minimum ellipticity at 193 nm representing the random state gradually shifted to 200 nm. As the ellipticity increased and started to form a positive peak at 200 nm, another minimum began to emerge at 220 nm characterizing the β-sheet structure of amyloid fibrils. Fully matured amyloid fibrils, therefore, produced the typical CD spectrum with a maximum peak at 200 nm and a minimum trough at 220 nm. Molar ellipticities at both 193 nm and 220 nm of each spectrum were compared with those of the spectrum obtained with the monomeric state. As the differences were formulated in – Δθ_193_/Δθ_220_ and plotted with the incubation period, the RAF forming α-synuclein obtained at 18.5 hr gave rise to the maximum value, from which mainly granular particles were revealed with an average size of 15 nm under TEM (**Figure 2D**). The RAF-producing oligomeric state can be evaluated as a state where the protein has lost its random characteristics but yet to form the β-sheet structure. It was thisβ-sheet free oligomeric state of α-synuclein that was responsible for the unit assembly leading to the RAF formation on the surface of lipid membranes.

**Figure 3:**
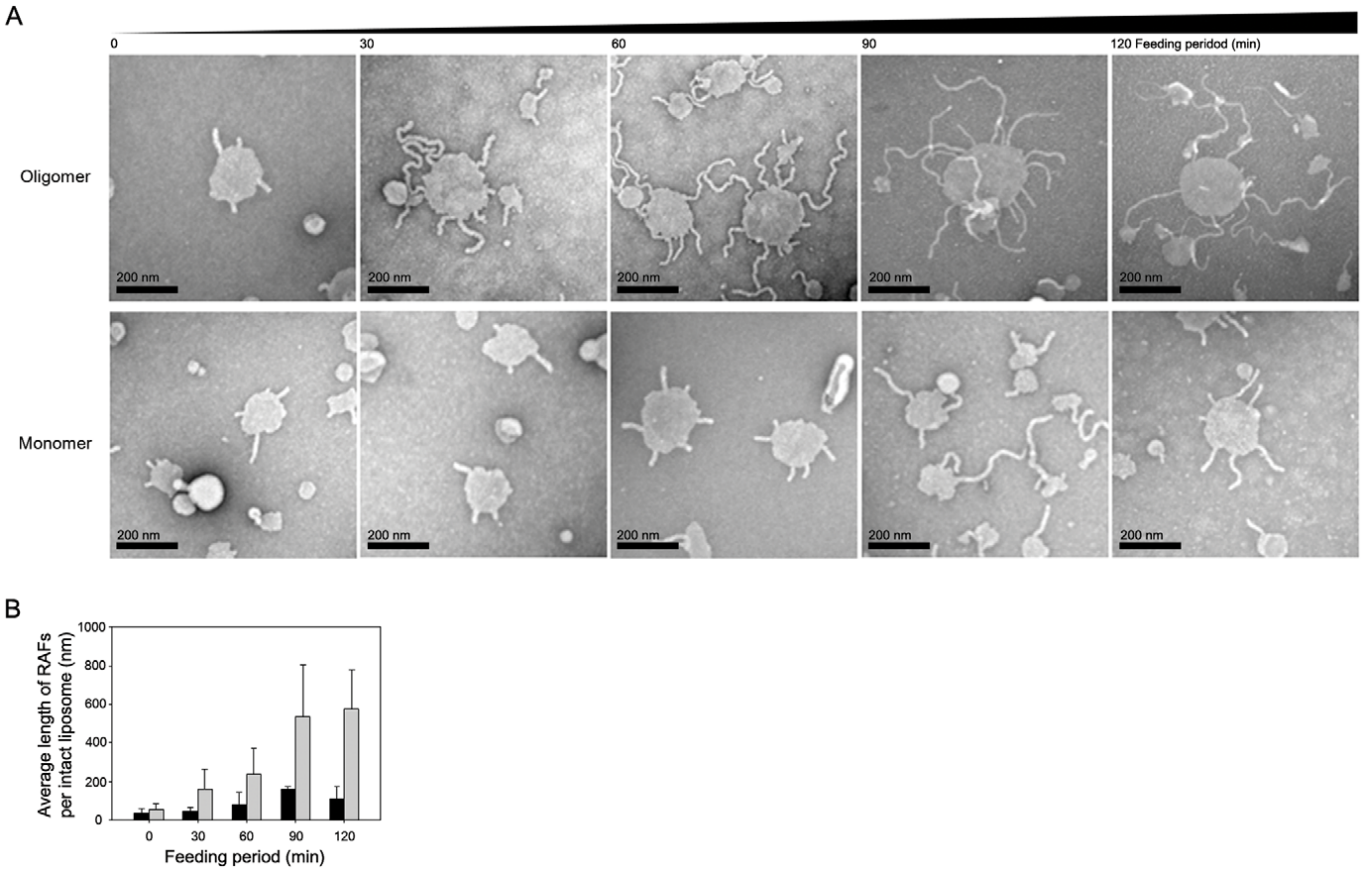
Maturation of the RAFs with either oligomers or monomers of α-synuclein. (**A**) The PC-liposomes with burgeoning RAFs prepared by incubating PC-liposomes (0.4 mg) with the oligomers (50 µg) in 20 mM Mes, pH 6.5, (0.25 ml) at room temperature for 5 min were sequentially fed with either the oligomers (upper panels) or the monomers (lower panels) of α-synuclein (50 µg per each addition) at every 30 min for total incubation of 2 hr under a quiescent incubation at 37°C. The fibrillar extension of RAFs was monitored with TEM. (B) Average lengths of RAFs were estimated by analyzing 200 separate PC-liposomes with RAFs at each time point. Gray and black bars indicate the average fibrillar lengths obtained with either oligomeric or monomeric form of α-synuclein, respectively.

In order to ensure whether RAFs could be further grown to larger suprastructures with elongated fibrils reminiscent of possible Lewy body structures, the PC-liposomes with burgeoning RAFs were matured by supplying additional α-synuclein in the form of either oligomers or monomers. The oligomers were able to elongate RAFs, which was far more effective than the fibrillar extension induced by the monomers (**Figure 3**). The oligomeric unit assembly actively proceeded for the initial part of the feeding procedure, which was followed by the membrane fragmentation/disintegration with a few liposomes still retaining the elongated RAFs on the surface (**upper panels in** **Figure 3A**, and **Figure S4**). This observation indicated that the oligomers were presumably added to the fibrils at the root interfaced with the surface of lipid membranes. In the case of the monomer feeding experiment, RAFs had been slowly extended for the entire 2 hr incubation (**lower panels in** **Figure 3A**). Average length of RAFs per intact liposome was substantially longer for the oligomer-induced RAFs over the monomer-treated cases (**Figure 3B**).

### Disruption of membrane structures with the RAF formation

**Figure 4:**
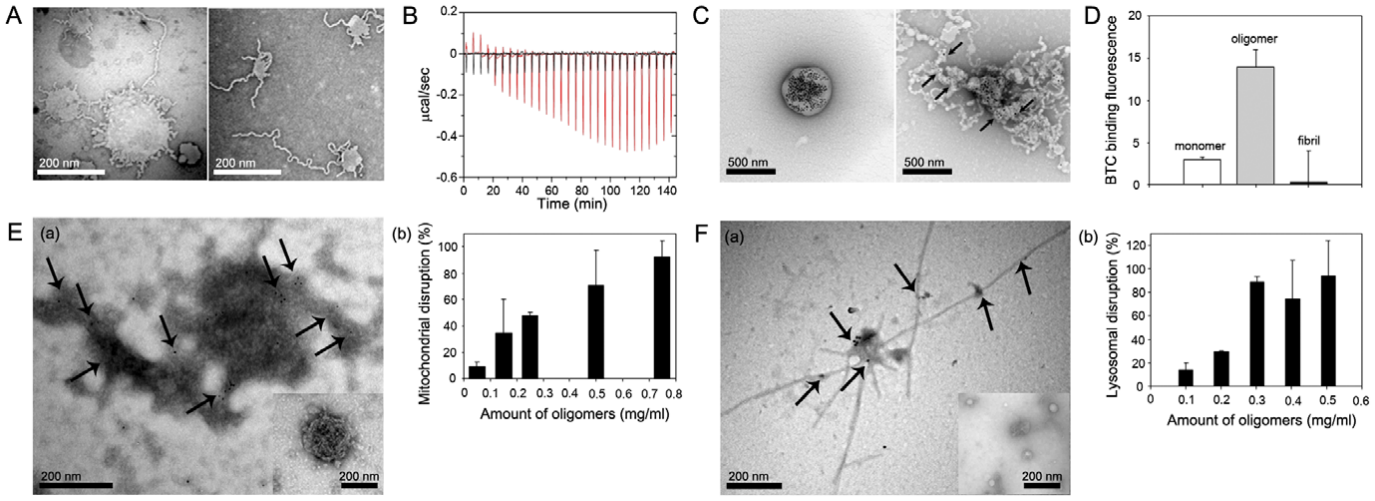
Disruption of lipid membranes upon the surface-dependent amyloid fibril formation. (**A**) PC-liposomes (1.25 mg/ml, 0.2 ml) revealed with TEM following a brief incubation in 20 mM Mes, pH 6.5, for 5 min at 25°C under a quiescent condition with the oligomeric α-synuclein at 220 µg (left panel) and 330 µg (right panel). (**B**) Thermodynamic assessment with isothermal titration calorimetry (ITC) for the molecular assembly-disassembly process of the PC-liposomes as treated with either the oligomers (red line) or the monomers (black line) of α-synuclein. The PC-liposomes (2.5 mg/ml, 1.7 ml) in 62.5 mM Mes, pH 6.5 were sequentially combined with α-synuclein in either form at 54.7 µg per addition for 20 sec at 5 min-interval. Negative value of y-axis represents exothermic reaction. (**C**) Disruption of the liposome containing magnetic nanoparticles (MNPs). The PC-liposomes enclosing the Fe_3_O_4_ magnetic nanoparticles were examined with TEM before (left) and after (right) the treatment of oligomeric α-synuclein at 0.5 mg/ml in 20 mM Mes, pH 6.5, for 5 min at 25°C. Arrows in the right panel indicate MNPs localized on the fibrils. (**D**) Calcium release from the Ca^2+^-entrapped PC-liposomes in the presence of monomers, oligomers, and amyloid fibrils of α-synuclein. The PC-liposomes containing 1 mM CaCl_2_ were incubated with monomers, oligomers, and fibrils of α-synuclein at 0.11 mg/ml in 20 mM Mes, pH 6.5, for 2 hr at 25°C. The release of Ca^2+^ ions was monitored with the Ca^2+^ ion-indicative fluorescent dye of BTC by detecting light emitting at 529 nm with an excitation at 401 nm. (**E and F**) Disruption of mitochondria (E) and lysosomes (F) with the oligomer treatment of α-synuclein. Mitochondria and lysosomes were visualized with TEM before (inset in a) and after (a) the oligomer treatment (0.5 mg/ml) in 20 mM Mes at pH 6.5 for 2 hr at 37°C. Presence of α-synuclein in the fibrils was confirmed with immunogold labeling with rabbit anti-α-synuclein antibody as indicated with arrows. Releases of lactate dehydrogenase (LDH) and cathepsin D were separately monitored upon the addition of oligomers with chromogenic substrates as a measure of disruption of mitochondria and lysosomes, respectively (b). Values are shown in means±s.d. (n = 2).

To elucidate a pathological implication of the physical structure formation of amyloid fibrils in cellular degeneration process, structural disruption of membranes was investigated upon the RAF formation on the membrane surface during the oligomeric unit assembly of α-synuclein (**Figure 4**). Dramatic shrinkage of liposomes was observed in addition to a few concentrically extended fibrils which remained attached to the membrane surface as the amount of oligomers was increased from 220 µg (**left panel in** **Figure 4A**) to 330 µg (**right panel in** **Figure 4A**). The surface-dependent oligomeric unit assembly and subsequent membrane disruption process was also evaluated with isothermal titration calorimetry (ITC) (**Figure 4B**). As a fixed amount of the oligomers (54.7 µg/addition) was consecutively added to PC-liposome (4.25 mg) at a speed of 20 sec/addition, the heat release (exothermic process) upon each addition increased gradually till 120 min following a brief period of endothermic process for initial 15 min, indicating that the oligomeric units appeared to be assembled on the lipid membranes (**red line in** **Figure 4B**). After passing the point of maximum heat release, the amount of heat released decreased, presumable suggesting that certain preformed structures started to be disrupted. Since the fibrils tended to be extended via the oligomeric unit assembly, the disrupting preformed structures would be the liposomes. When monomeric α-synuclein was serially added to the membranes, however, the amount of heat released upon each addition remained almost unaffected (**black line in** **Fig. 4B**). These data indicated that the oligomers were capable of promoting dramatic structural transitions in the liposomes through molecular assembly-disassembly process as demonstrated by the amyloid fibril extension and the lipid membrane disruption.

In order to portray actual membrane disruption process, magnetic nanoparticles (MNPs) were selectively enclosed within PC-liposomes. When the oligomeric species were allowed to interact with the MNP-containing liposomes, the fibrils were developed by carrying MNPs as the membranes were disintegrated (**Figure 4C**). The lipid molecules appeared to be pulled away from the membranes to the extended fibrils, which resulted in the membrane disruption and the fibrillar extension with ragged surface retaining MNPs. The liposomal shrinkage occurred concomitantly with the fibrillar extension by consuming the lipid molecules. In other words, the requirement of lipid molecules for the fibrillar extension would inadvertently lead to the membrane disintegration. The membrane disruption was also probed with calcium release assay from calcium-loaded liposomes. As the liposomes were treated with monomers, oligomers, and amyloid fibrils of α-synuclein at 0.11 mg/ml in 20 mM Mes, pH 6.5, the amount of calcium released from the liposomes was monitored with the coumarin benzothiazole-based Ca^2+^ indicator (BTC) present outside the membranes. While the monomeric α-synuclein and the preformed amyloid fibrils failed to release Ca^2+^ ions, only the oligomeric forms were demonstrated to be capable of releasing the calcium (**Figure 4D**).

To find pathological relevance of the membrane disruption via the oligomer-mediated amyloid fibril formation, the potential intracellular toxic depots of mitochondria and lysosomes were tested for their membrane destabilization with the oligomeric species of α-synuclein. Following the treatment of oligomers, mitochondria isolated from Hela cell were disintegrated with the formation of dark extensions where the presence of α-synuclein was confirmed with immunogold staining as indicated with arrow heads in **Figure 4E**. The fibril formation upon the mitochondrial disintegration was not as clear as the RAFs obtained with liposomes (**Figure 4E** **-a**), which could be due to heterogeneity of proteins and lipids comprising mitochondria. The organelle disruption was assessed with the release of lactate dehydrogenase as a marker enzyme of mitochondria, which increased proportionally with the amount of oligomeric α-synuclein treated (**Figure 4E** **-b**). The fibril formation on the lysosomes obtained from rat liver, on the other hand, was discrete as shown in **Figure 4F**. α-Synuclein was also confirmed to comprise the fibrils with the immunogold staining as they formed on the periphery of lysosomes (**Figure 4F** **-a**). The release of a lysosomal enzyme cathepsin D was also linear with the amount of oligomeric α-synuclein treated (**Figure 4F** **-b**). Disruption of the pathologically critical organelles of mitochondria and lysosomes upon the amyloid fibril formation through the oligomeric unit assembly process, therefore, ought to be devastating to the cells.

## Discussion

Double-concerted fibrillation mechanism we have proposed on the basis of the accelerated amyloid fibrillation of α-synuclein from its oligomeric state in the presence of organic solvent, pH change, and shear force has been demonstrated to operate in the RAF formation on the surface of lipid membranes through the unit assembly process (**Figure S5**) [4]–[6], [11]. Not all the oligomeric species, however, were able to produce RAFs on the membrane surface. The RAF-forming oligomers had to satisfy the prerequisite of subtle structural rearrangement to exhibit the unit assembly. The β-sheet free oligomeric species of α-synuclein was specifically shown to exist in a pseudo-stable state which could be readily altered to turn into the suprastructure of amyloid fibrils at the lipid membrane surface. Combining with the membrane destabilization activity of the RAF formation, the unit assembly process of the oligomeric species can be appreciated for the eventual development of controlling strategies toward PD by targeting the RAF-forming oligomeric species.

Lewy bodies are heterogeneous intracellular inclusions classified into either concentric or homogeneous (irregular) form [10]. Classical concentric Lewy bodies (LBs) are spherical inclusions which consist of a peripheral halo made of radiating filaments of α-synuclein and a dense core where lipids are found in vesicular profiles [12]. The other group of homogeneous LBs show irregular morphology as originally depicted by Lewy in 1912, which comprise randomly oriented fibrils and a large number of embedded mitochondria [13]. It was speculated that the irregular LBs could be developed into the classical LBs [10]. Since those RAF-containing structures obtained with either lysosomes and mitochondria have been demonstrated to be distinctive in this study, the heterogeneous LBs found in pathological conditions could have been developed into mutually independent pools in the presence of the intracellular membranous structures.

The RAF formation and subsequent fibrillar extension in the presence of continuous supply of both monomeric and oligomeric forms of α-synuclein under our simplified *in vitro* system could be considered as a basic process to reach the LB structures *in vivo*. During the LB maturation, other components such as neuromelanin, lipofuscin, organelles including mitochondria and dense core vesicles, and amorphous materials are admixed with the developing fibrils which also experience biochemical modifications including phosphorylation, ubiquitination, proteolysis, and oxidative crosslinking [14], [15]. In particular, the RAF extension phenomenon by pulling lipids away from the membranes could support an observation of the coexistence of α-synuclein and lipids in the peripheral part of LB although most lipids are concentrated at the core region [16], [17]. The α-synuclein-mediated membrane disintegration has also been noticed in the other recent studies [18], [19]. The immature RAF-containing structures could be, therefore, enlarged to mature LBs through various biochemical maturation processes. Alternatively, the RAF-containing structures could vary their compositions and physical sizes depending on initial membrane states which provide a tethering spot for the RAF formation. The RAF-burgeoning membrane structures obtained by coincubating the α-synuclein oligomers and the lipid membranes such as synthetic liposomes, mitochondria, and lysosomes are thus hypothesized to be previously unrecognized suprastructures which could develop into mature LBs under pathological conditions.

Since the discovery of LBs, they have been considered as a pathological component of Parkinson's disease. Their involvement in the neuronal cell death, however, has been depreciated since those structures have been found even in the healthy neurons, and thus considered as inert detoxification end product [20], [21]. Nonetheless, the data obtained in this study also suggest that the LB formation process *per se* could be a toxic mechanism affecting cell viability by influencing the membrane integrity of intracellular organelles [18]. In addition, the resulting LBs might be a product of the toxic event of membrane disruption process rather than the detoxification product. Unlike the previous studies counting amyloid fibrils as an inert detoxification end product or a passive matrix to produce toxic substances [20]–[23], active role of amyloid fibril formation on the cellular degeneration has been proposed in this study as the fibrillation on the membrane surface through the oligomeric unit assembly process could contribute to the neuronal degeneration by disrupting intracellular membrane structures which release potential toxic substances responsible for the cell death. In fact, membrane damage due to the lipid extraction by the amyloid fibril formation on the surface of lipid membranes was recently suggested to be a cause for the neuronal degeneration observed with α-synuclein [18]. Besides the amyloid fibril formation, the acidic lipid membranes especially made of phosphatidylglycerol could also be disrupted via the α-synuclein-mediated membrane tubulation process [19]. These physical structure formations and thus exhibiting mechanical effects on the membrane stability, therefore, could be considered as an alternative toxic mechanism to the prevalent general notion of chemical causes for cell death.

## Materials and Methods

### Protein purification

Briefly, the heat-treated (100°C for 20 min) cell lysate of *E. coli* overexpressing human α-synuclein was centrifuged [24]. Then the supernatant was loaded onto DEAE-Sephacel anion-exchange column pre-equilibrated with 20 mM Tris-Cl, pH 7.5, containing 0.1 M NaCl, and eluted with 0.4 M NaCl under a linear gradient. Subsequently, Sephacryl S-200 size-exclusion chromatography was performed with 20 mM Mes, pH 6.5, containing 0.1% NaN_3_. The α-synuclein-containing fractions were combined and applied to S-Sepharose cation-exchange chromatography equilibrated with 20 mM Mes, pH 6.5. All the chromatographic processes were performed at 4°C. The protein was eluted with NaCl. The purified synuclein was dialyzed against 20 mM Mes, pH 6.5, and diluted to 1 mg/ml. It was then stored at −20°C in aliquots.

### Fibrillation of α-synuclein and preparation of oligomeric species

The protein (1 mg/ml) was fibrillated in 20 mM Mes, pH 6.5, at 37°C under continuous shaking incubation at 200 rpm with an orbit shaker (Vision Scientific Co, Bucheon, Korea). To follow the fibrillation process, the aggregation kinetics was monitored with ThT assay (**Figure 2A** and **Figure S1B**), and the resulting protein aggregates were examined with transmission electron microscope (EF-TEM; Carl Zeiss, Munich, Germany) (**inset in Figure S3**). Unless otherwise mentioned, the oligomeric α-synuclein was collected at a time point of 18.5 hr during the fibrillation carried out at 37°C under a shaking incubation at 200 rpm.

### Thioflavin-T assay

To monitor the aggregation kinetics, an aliquot (20 µl) of the protein during the incubation was combined with ThT (2.5 µM) in 50 mM glycine, pH 8.5, to a final volume of 200 µl. The ThT binding fluorescence at 482 nm with excitation at 450 nm was measured with LS-55 luminescence spectrophotometer (Perkin-Elmer, Waltham, MA). To evaluate the formation of the radiating amyloid fibrils (RAFs) on lipid membrane, either form of monomeric or oligomeric α-synuclein was combined with PC-liposome (0.57 mg/ml) and ThT (2.5 µM) in 50 mM glycine, pH 8.5, as the protein concentration was increased from 0 to 0.23 mg/ml (**Figure 1C**). The net value of ThT binding fluorescence was calculated by subtracting the fluorescence intensity of the sample obtained without PC-liposome from that with PC-liposome.

### Energy-filtering transmission electron microscope (EF-TEM)

The aggregates of protein were visualized with energy-filtering transmission electron microscope. Samples containing monomer, oligomer or fibrils of α-synuclein (20 µl) were adsorbed onto a carbon-coated copper grid (200 mesh) and air dried. An negative staining of samples was performed with 2% uranyl acetate (Electron Microscopy Sciences, Hatfield, PA) for one minute, and the protein aggregates were examined with EF-TEM.

### Liposome synthesis

L-α-phosphatidylcholine (PC; Sigma, St. Louis, MO) was dissolved with chloroform at 2.5 mg/ml in a test tube, and followed by blowing N_2_ gas until chloroform removed completely. The tube was stored in −20°C for 1 hr, and it was rapidly shifted into a water bath of 60°C to peel the lipid film readily. This film was rehydrated in 1 ml of 20 mM Mes at pH 6.5. The suspension was extruded at 65°C using an extruder set with 400 nm pore-sized polycarbonate membrane (Avanti Polar Lipids mini-extruder, Northern Lipids Inc., Burnaby, BC, Canada). The homogeneous suspension of unilamellar vesicles was examined with EF-TEM (**Figure S1**).

### Congo red staining

The oligomers (100 µg) was combined with PC-liposome (2.5 mg/ml, 100 µl), and followed by a standing incubation at 37°C for 18 hr to complete the formation of the RAF-containing aggregates. After an aliquot (50 µl) was placed on a Si wafer slice and air-dried for 30 min at 60°C, the sample was stained with 1% congo red in 80% ethanol for 30 min. For washing, the sample was sequentially dipped in distilled water, 80% ethanol, and absolute ethanol for a few seconds each. Birefringence of the sample was observed with a polarized microscope (**Figure 1B**).

### Circular Dichroism (CD)

α-Synuclein (1 mg/ml, 100 µl) in either state of monomers or oligomers was combined with PC-liposome (2.5 mg/ml, 100 µl), and followed by a standing incubation at 37°C for 18 hr to complete the RAF aggregate formation (**Figure 1D**). As controls, the lipid-free monomers and oligomers (0.5 mg/ml, 200 µl) were also prepared through the quiescent incubation at 37°C for 18 hr (**Figure S2**). The spectra were measured within a 0.1 mm-pathlength quartz cuvette between 195 and 250 nm with a step resolution of 1.0 nm and scan speed of 20 nm/min. All of the spectra were obtained from an average of five separate scans. In addition, structural transition of α-synuclein during the fibrillation was also assessed with CD spectroscopy (**Figure 2C**). While the protein (1 mg/ml) was incubated at 37°C with a shaking condition at 200 rpm, aliquots of the solution were collected at various time points shown in **Figure 2A** and stored at −20°C before their CD analyses under the same monitoring conditions as described above.

### Isothermal Titration Calorimetry (ITC) study

α-Synuclein (1.75 mg/ml, 1 ml) were dried for more than 10 hr using a freeze dryer (Eyela FDU-2200, Tokyo Rikakikai Co., Tokyo, Japan), and then the protein powder was resuspended in 0.32 ml of distillated water. The resulting protein samples were degassed at 25°C for 20 min to avoid bubble formation during stirring. Titration of the protein into PC-liposome (3.26 mM in 62.5 mM Mes pH 6.5) was performed at 25°C using ITC (VP-ITC, Microcal LLC, Northampton, MA). During the titration, aliquots of 10 µl were added into the PC-liposome solution for 20 sec in a calorimeter at 300 sec-interval, and the heat released with each injection was measured (**Figure 4B**).

### Liposomes containing magnetic nanoparticles (MNPs)

Iron oxide nanoparticles (Fe_3_O_4_) were synthesized by previously reported method without modification [25]. Then, MNPs embedded liposomes were prepared by the method described previously [26] with some modifications. Briefly, MNPs (25 mg/ml, 0.1 ml) was mixed with PC lipids (2.5 mg/ml, 0.8 ml) in chloroform. After evaporating solvent, the mixture was incubated at 60°C under vacuum for 1 hr. The addition of 20 mM Mes, pH 6.5, (1 ml) resulted in the nanoparticle-containing liposomes. Excess PC lipids were removed by ultracentrifugation. To study the interaction between oligomeric α-synuclein and Fe_3_O_4_-PC lipid complexes, the oligomer (0.05 mg) was combined with the Fe_3_O_4_-PC lipid complex suspension (50 µl). After an incubation for 5 min at room temperature, the migration of nanoparticles in the core region of the Fe_3_O_4_-PC lipid complex was observed with EF-TEM.

### Calcium release assay from the calcium-loaded liposome

The PC lipid film was prepared as described above, and was rehydrated in 1 ml of 20 mM Mes, pH 6.5, containing 1 mM CaCl_2_. The suspension of multilamellar vesicles was extruded with 400 nm pore-sized membrane. The resulting unilamellar vesicle of PC containing Ca^2+^ was dialyzed twice in 20 mM Mes, pH 6.5, without CaCl_2_ at 4°C for 4 hr by using Slide-A-Lyzer Dialysis Cassette G2 (Thermo Scientific, Waltham, MA). Then, the Ca^2+^ ion-indicative fluorescent dye, BTC (tetrapotassium salt form; Invitrogen, Carlsbad, CA) was used to monitor the stability of the lipid membrane as the liposomes were treated with α-synuclein in monomeric, oligomeric, or amyloid fibrillar form. The PC-liposome containing Ca^2+^ (100 µl) was combined with BTC (60 µM, 50 µl) and then the sample solution was incubated with α-synuclein (1 mg/ml, 20 µl). The BTC fluorescence intensity at 529 nm was detected with an excitation at 401 nm through using LS-55 luminescence spectrophotometer (Perkin-Elmer, Waltham, MA).

### Isolation of mitochondria and lysosomes

According to the procedures provided by the manufacture, mitochondria were isolated from murine liver and Hela cells by using mitochondria isolation kit for tissue (#89801, Pierce, Rockford, IL) and mitochondria isolation kit for cultured cells (#89874, Pierce, Rockford, IL), respectively. In brief, murine liver was minced into small pieces and Dounce-homogenized (70–80 strokes for 200 mg) with a glass grinder on ice. Hela cells (2×10^7^) were harvested by a centrifugation (850×g, min). Mitochondria were isolated from either tissue extracts or cells through successive centrifugation steps after adding the reagents of the kits. To isolate lysosomes from murine liver, lysosome enrichment kit for tissue and cultured cells (#89839, Pierce, Rockford, IL) was used. The tissue extracts were prepared from murine liver by the same method used for the isolation of mitochondria. Then, we performed a density gradient centrifugation (145,000×g for 2 hours at 4°C) through a discontinuous density gradient by overlaying the provided gradient solutions. Lysosomes were obtained in the top band of the gradient. Lysosomes were further purified through the successive centrifugation steps. The isolated organelles were examined and assessed with EF-TEM. These organelles were stored at 4°C before their use.

### Mitochondrial disruption

Mitochondrial disruption induced by the RAF formation was evaluated with the release of lactate dehydrogenase (LDH). The LDH release was detected with the LDH cytotoxicity detection kit (#11644793001, Roche, Germany) according to the protocol with some modifications. Briefly, mitochondria (20 µl) obtained from Hela cells were combined with various concentrations of the oligomeric α-synuclein (20 µl), to a final volume of 200 µl in 50 mM Tris-HCl, pH 7.5. After a standing incubation at 37°C for 1 hr, the media were collected, and added into freshly prepared reaction mixture (100 µl) in 96-well microtitre plate. The amount of LDH released was measured at 492 nm by a BioTek EL-340 microplate reader. Maximum LDH release was determined by adding 5% Trition-X 100 (20 µl) in the absence of α-synuclein (high control). Spontaneous LDH release was also detected by adding 20 mM Mes, pH 6.5, (20 µl) in the absence of α-synuclein (low control). Degree of membrane disruption was calculated in percentage by using the following equation. 
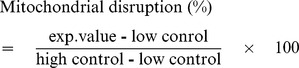



All experiments were performed in triplicate.

### Lysosomal disruption

To evaluate the lysosomal disruption induced by the RAF formation, cathepsin D release was analyzed with the cathepsin D assay kit (Sigma, St. Louis, MO). The lysosome obtained from murnie (50 µl) was treated with various concentrations of the oligomeric α-synuclein (50 µl) in 20 mM Mes, pH 6.5. After a standing incubation at 37°C for 1 hr to induce the RAF formation, cathepsin D substrate solution (340 µM, 50 µl) was added to the sample solution. After an additional standing incubation at 37°C for 30 min, the amount of cathepsin D released from each sample was measured with fluorescence intensity at 393 nm with an excitation at 328 nm. Maximum cathepsin D release was determined by sonication for 30 sec in the absence of α-synuclein (high control). Spontaneous cathepsin D release was also detected by adding 20 mM Mes, pH 6.5, (50 µl) in the absence of α-synuclein (low control). The lysosomal disruption was calculated by using the following equation. 
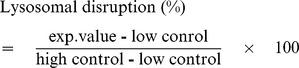



All experiments were performed in triplicate.

### Immuno-gold labeling

The mitochondria or lysosomes prepared from murine liver were mixed with the oligomeric α-synuclein (1 mg/ml, 50 µl), and incubated at 37°C for 1 hr. An aliquot (20 µl) of the samples was adsorbed onto copper grid, and incubated at room temperature for 2 hr in 2 µg/ml goat polyclonal anti-α-synuclein antibody (Santa Cruz Biotechnology, Santa Cruz, CA). After washing with PBS, the grids were incubated for 2 hr in 10-nm gold rabbit anti-goat conjugate (Ab 27245, Abcam, Cambridge, UK). After an additional washing and a negative staining with 2% uranyl acetate, the microscopic images were visualized with EF-TEM.

## Supporting Information

Figure S1 **Preparation of PC-liposomes and oligomeric α-synuclein.** **(A) **PC-liposome (200 nm) was prepared by the extrusion method. **(B) **α-Synuclein (1 mg/ml) in 20 mM Mes, pH 6.5 was incubated at 37°C with agitation at 600 rpm, and their aggregation kinetics was followed by thioflavin-T binding fluorescence. The oligomeric intermediates of the protein were collected at 8 hr during the fibrillation (black arrow). The morphology of the collected oligomers was observed with EF-TEM (inset).
(TIF)

Figure S2 **CD spectra of monomeric α-synuclein before and after the incubation with PC-liposomes. **CD spectra of monomeric α-synuclein were monitored as control experiment for **Figure 1D** **. Black and red dots indicate the CD spectra of α-synuclein before and after a standing incubation with PC-liposomes at 37°C for 18 hr, respectively.**
(TIF)

Figure S3 **RAF formation between PC-liposomes and various oligomeric species of α-synuclein. **To characterize the oligomeric species responsible for the RAF formation, the protein aggregates were collected at various specific time points during the fibrillation process under a shaking incubation (200 rpm) at 37°C. The suprastructure formation was examined with EF-TEM. The inset images show the morphologies of the protein aggregates collected at the time points in the absence of PC-liposomes.
(TIF)

Figure S4 **Various TEM images of the disrupted liposomes with α-synuclein oligomers. **PC liposomes (0.25 mg) were incubated with the α-synuclein oligomers (100 µg) in 20 mM Mes, pH 6.5, at room temperature for 5 min.
(TIF)

Figure S5 **Illustrative scheme of the RAF formation on the surface of liposomes. (A)** Double-concerted model explaining the amyloid fibril formation via unit-assembly process of the oligomeric α-synuclein. Soluble monomeric α-synuclein aggregates and forms the β-sheet free oligomers. As these pseudo-stable oligomers sense environmental factors including shear force, organic solvent, and the hydrophilic-hydrophobic interface of lipid membranes, they become more aggregative oligomers with concomitant internal structure rearrangement, which results in the accelerated amyloid fibril formation. **(B)** Formation of radiating amyloid fibrils (RAFs) on the surface of liposomes. The β-Sheet free oligomers would experience the structural rearrangement at the interface of lipid membranes, and they subsequently form the RAFs on the surface of liposomes. The RAFs are further elongated to form the Lewy-body like (LBL) structure as they are incubated with the oligomeric α-synuclein.
(TIF)

## References

[1] Chiti F, Dobson CM (2006) Protein misfolding, functional amyloid, and human disease. Annu Rev Biochem 75: 333–366. 16756495 10.1146/annurev.biochem.75.101304.123901

[2] Ross CA, Poirier MA (2004) Protein aggregation and neurodegenerative disease. Nat Med 10: S10–S17. 15272267 10.1038/nm1066

[3] Hardy J, Selkoe DJ (2002) The amyloid hypothesis of Alzheimer's disease: Progress and problems on the road to therapeutics. Science 297: 353–356. 12130773 10.1126/science.1072994

[4] Lee J-H, Bhak G, Lee S-G, Paik SR (2008) Instantaneous amyloid fibril formation of α-synuclein from the oligomeric granular structures in the presence of hexane. Biophys J 95: L16–L18. 18469076 10.1529/biophysj.108.135186 PMC2440477

[5] Bhak G, Lee J-H, Hahn J-S, Paik SR (2009) Granular assembly of α-synuclein leading to the accelerated amyloid fibril formation with shear stress. PLoS ONE 4: e4177. 19137068 10.1371/journal.pone.0004177 PMC2613562

[6] Lee D, Choe Y-J, Choi YS, Bhak G, Lee J, et al (2011) Photoconductivity of pea-pod-type chains of gold nanoparticles encapsulated within dielectric amyloid protein nanofibrils of α-synuclein. Angew Chem Int Ed Engl 50: 1332–1337. 21290506 10.1002/anie.201004301

[7] Spillantini MG, Schmidt ML, Lee VM-Y, Trojanowski JQ, Jakes R, et al (1997) α-synuclein in Lewy bodies. Nature 388: 839–840. 9278044 10.1038/42166

[8] Spillantini MG, Crowther RA, Jakes R, Hasegawa M, Goedert M (1998) α-Synuclein in filamentous inclusions of Lewy bodies from Parkinson's disease and dementia with Lewy bodies. Proc Natl Acad Sci USA 95: 6469–6473. 9600990 10.1073/pnas.95.11.6469 PMC27806

[9] Masliah E, Rockenstein E, Veinbergs I, Mallory M, Hashimoto M, et al (2000) Dopaminergic loss and inclusion body formation in α-synuclein mice: Implications for neurodegenerative disorders. Science 287: 1265–1269. 10678833 10.1126/science.287.5456.1265

[10] Gai WP, Yuan HX, Li XQ, Power JTH, Blumbergs PC, et al (2000) In situ and in vitro study of colocalization and segregation of α-synuclein, ubiquitin, and lipids in Lewy bodies. Exp Neur, N Y 166: 324–333. 10.1006/exnr.2000.7527 11085897

[11] Bhak G, Choe Y-J, Paik SR (2009) Mechanism of amyloidogenesis: Nucleation-dependent fibrillation versus double-concerted fibrillation. BMB Rep 42: 541–551. 19788854 10.5483/bmbrep.2009.42.9.541

[12] Forno LS (1996) Neuropathology of Parkinson's disease. J Neuropathol Exp Neurol 55: 259–272. 8786384 10.1097/00005072-199603000-00001

[13] Roy S, Wolman L (1969) Ultrastructural observations in Parkinsonism. J Pathol 99: 39–44. 5359222 10.1002/path.1710990106

[14] Hasegawa M, Fujiwara H, Nonaka T (2002) Phosphorylated α-synuclein is ubiquitinated in α-synucleinopathy lesions. J Biol Chem 277: 49071–49076. 12377775 10.1074/jbc.M208046200

[15] Giasson BI, Duda JE, Murray IVJ, Chen Q, Souza JM, et al (2000) Oxidative damage linked to neurodegeneration by selective α-synuclein nitration in synucleinopathy lesions. Science 290: 985. 11062131 10.1126/science.290.5493.985

[16] McNaught KSP, Olanow W (2006) Protein aggregation in the pathogenesis of familial and sporadic Parkinson's disease. Neurobiol Aging 27: 530–545. 16207501 10.1016/j.neurobiolaging.2005.08.012

[17] Shults CW (2006) Lewy bodies. Proc Natl Acad Sci USA 106: 1661–1668. 10.1073/pnas.0509567103 PMC1413649 16449387

[18] Reynolds NP, Soragni A, Rabe M, Verdes D, Liverani E, et al (2011) Mechanism of membrane interaction and disruption by α-synuclein. J Am Chem Soc 133: 19366–19375. 21978222 10.1021/ja2029848

[19] Varkey J, Isas JM, Mizuno N, Jensen MB, Bhatia VK, et al (2010) Membrane curvature induction and tubulation are common features of synucleins and apolipoproteins. J Biol Chem 285: 32486–32493. 20693280 10.1074/jbc.M110.139576 PMC2952250

[20] Conway KA, Lee S-J, Rochet J-C, Ding TT, Williamson RE, et al (2000) Acceleration of oligomerization, not fibrillation, is a shared property of both α-synuclein mutations linked to early-onset Parkinson's disease: Implications for pathogenesis and therapy. Proc Natl Acad Sci USA 97: 571–576. 10639120 10.1073/pnas.97.2.571 PMC15371

[21] Goldberg MS, Lansbury PT (2000) Is there a cause-and-effect relationship between α-synuclein fibrillization and Parkinson's disease? Nat Cell Biol 2: E115–E119. 10878819 10.1038/35017124

[22] Huang X, Cuajungco MP, Atwood CS, Hartshorn MA, Tyndall JDA, et al (1999) Cu(II) potentiation of Alzheimer Aβ neurotoxicity. J Biol Chem 274: 37111–37116. 10601271 10.1074/jbc.274.52.37111

[23] Bush AI, Tanzi RE (2002) The galvanization of β-amyloid in Alzheimer's disease. Proc Natl Acad Sci USA 99: 7317–7319. 12032279 10.1073/pnas.122249699 PMC124227

[24] Paik SR, Lee J-H, Kim D-H, Chang C-S, Kim J (1997) Aluminum-Induced Structural Alterations of the Precursor of the Non-Aβ Component of Alzheimer's Disease Amyloid Arch Biochem Biophys. 344: 325–334. 10.1006/abbi.1997.0207 9264546

[25] Park J, An K, Hwang Y, Park J-G, Noh H-J, et al (2004) Ultra-large-scale syntheses of monodisperse nanocrystals. Nat Mater 3: 891–895. 15568032 10.1038/nmat1251

[26] Dubertret B, Skourides P, Norris DJ, Noireaux V, Brivanlou AH, et al (2002) In vivo imaging of quantum dots encapsulated in phospholipid micelles. Science 298: 1759–1762. 12459582 10.1126/science.1077194

