# Supplementary figures and images for: Radiating Amyloid Fibril Formation on the Surface of Lipid Membranes through Unit-Assembly of Oligomeric Species of α-Synuclein
Source: PLoS One. 2012 Oct 15;7(10):e47580. doi: 10.1371/journal.pone.0047580 (PMC3471876; doi:10.1371/journal.pone.0047580)

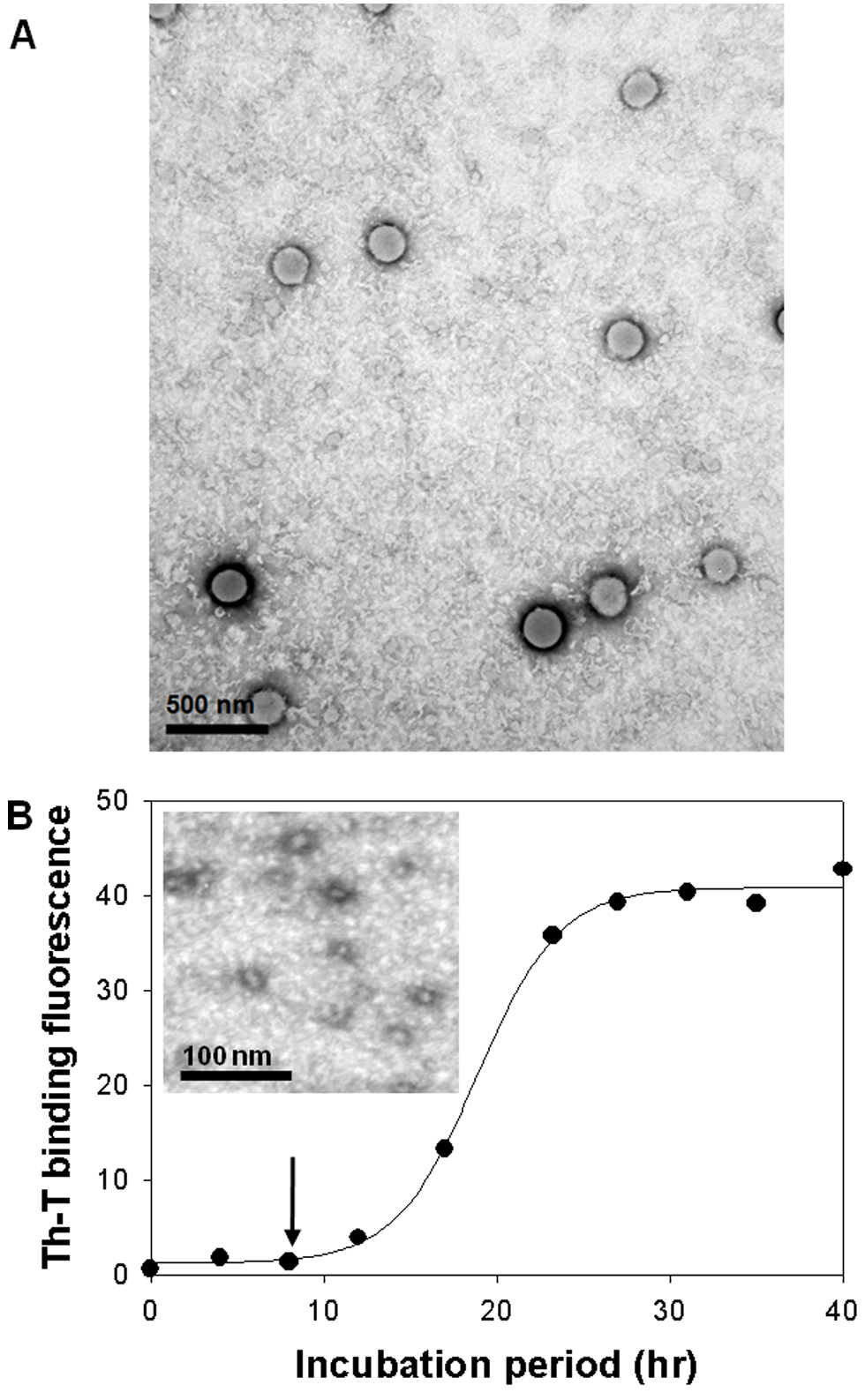

Supplement: Figure S1 — Preparation of PC-liposomes and oligomeric α-synuclein. (A) PC-liposome (200 nm) was prepared by the extrusion method. (B) α-Synuclein (1 mg/ml) in 20 mM Mes, pH 6.5 was incubated at 37°C with agitation at 600 rpm, and their aggregation kinetics was followed by thioflavin-T binding fluorescence. The oligomeric intermediates of the protein were collected at 8 hr during the fibrillation (black arrow). The morphology of the collected oligomers was observed with EF-TEM (inset). (TIF) [file pone.0047580.s001.tif]

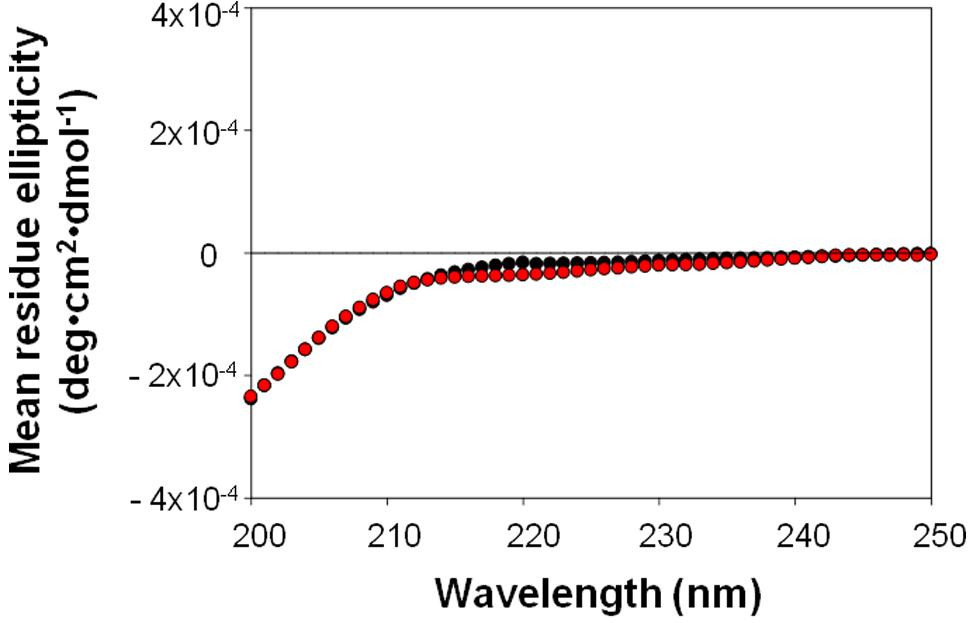

Supplement: Figure S2 — CD spectra of monomeric α-synuclein before and after the incubation with PC-liposomes. CD spectra of monomeric α-synuclein were monitored as control experiment for Figure 1D . Black and red dots indicate the CD spectra of α-synuclein before and after a standing incubation with PC-liposomes at 37°C for 18 hr, respectively. (TIF) [file pone.0047580.s002.tif]

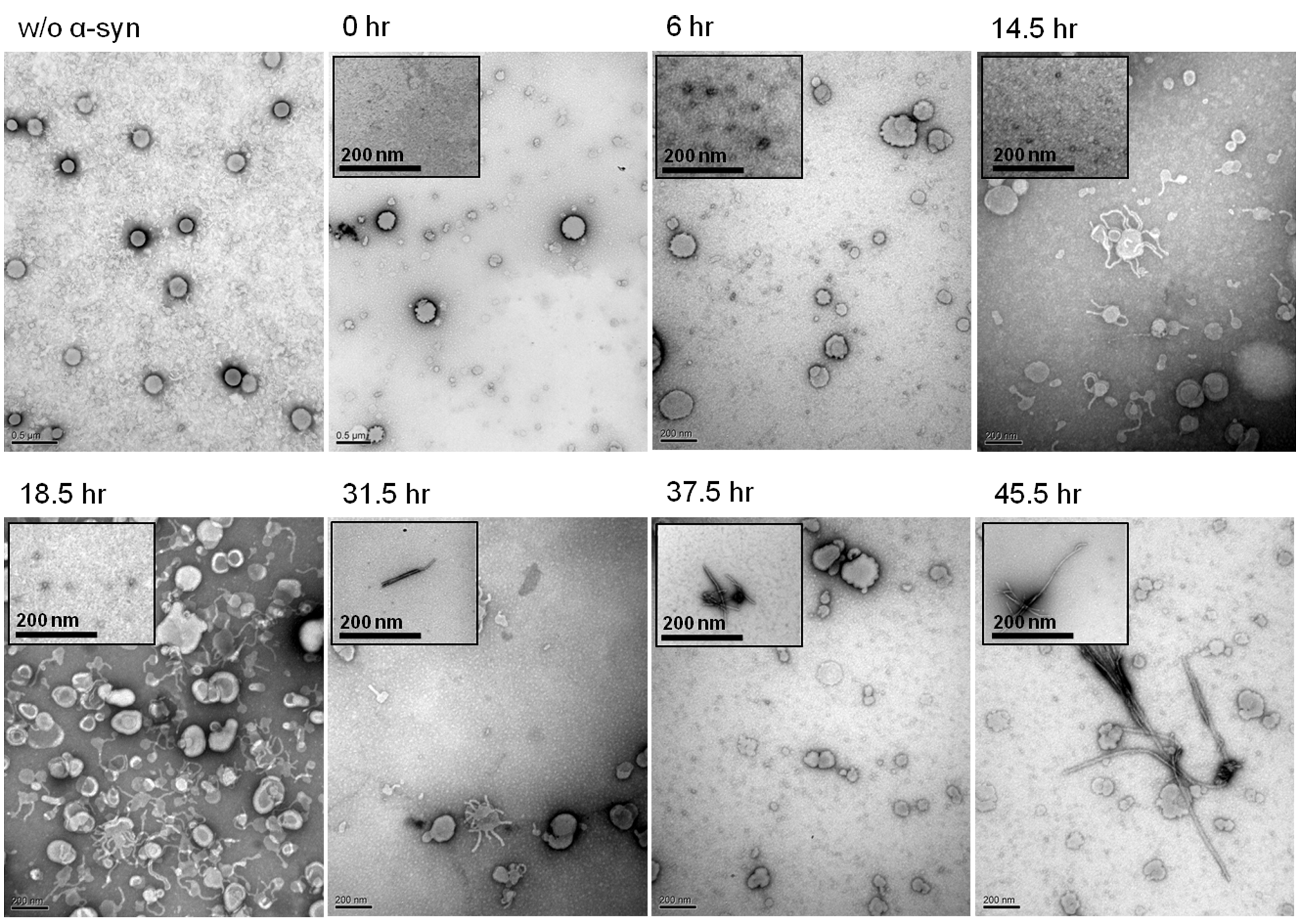

Supplement: Figure S3 — RAF formation between PC-liposomes and various oligomeric species of α-synuclein. To characterize the oligomeric species responsible for the RAF formation, the protein aggregates were collected at various specific time points during the fibrillation process under a shaking incubation (200 rpm) at 37°C. The suprastructure formation was examined with EF-TEM. The inset images show the morphologies of the protein aggregates collected at the time points in the absence of PC-liposomes. (TIF) [file pone.0047580.s003.tif]

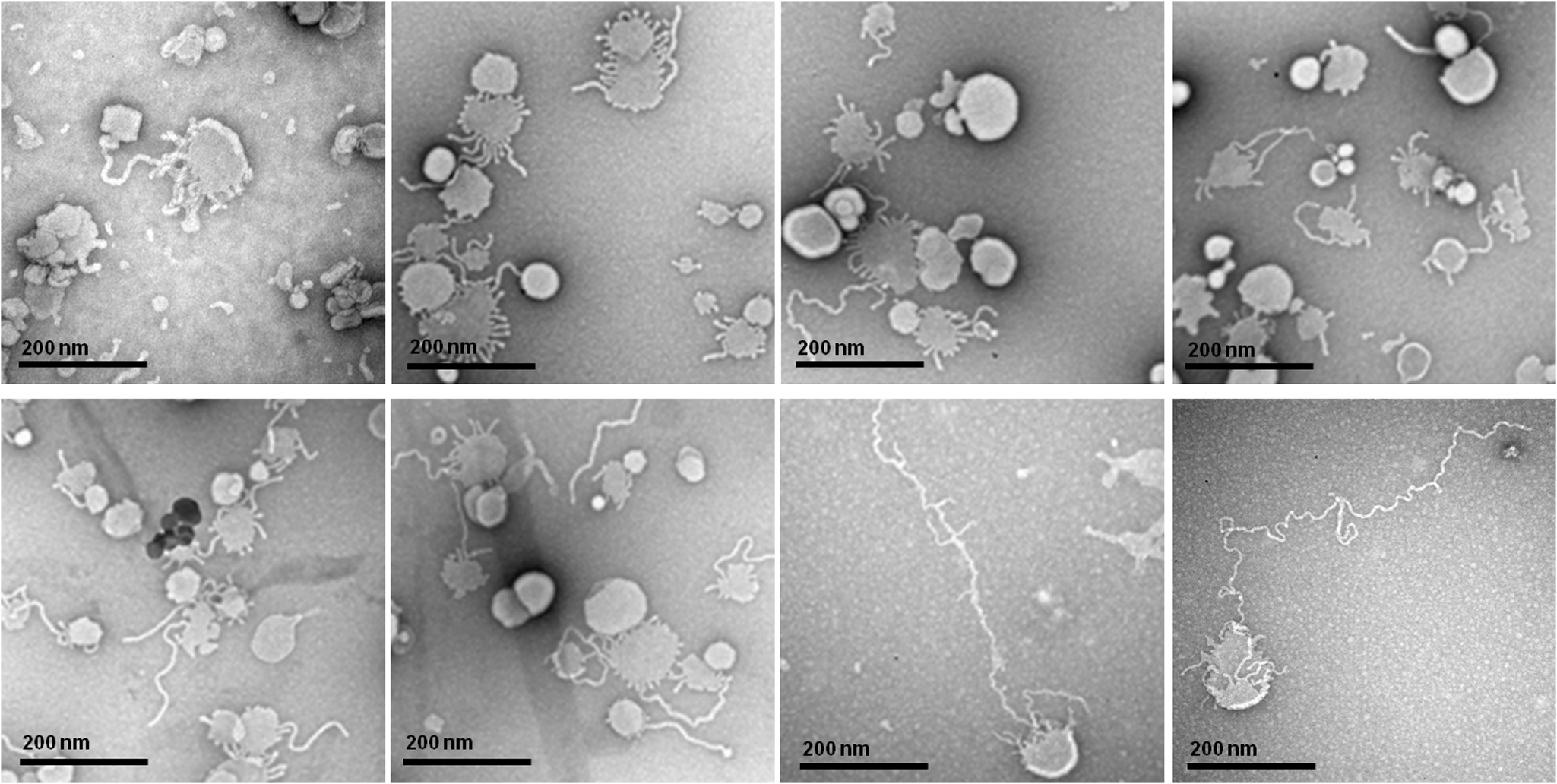

Supplement: Figure S4 — Various TEM images of the disrupted liposomes with α-synuclein oligomers. PC liposomes (0.25 mg) were incubated with the α-synuclein oligomers (100 µg) in 20 mM Mes, pH 6.5, at room temperature for 5 min. (TIF) [file pone.0047580.s004.tif]

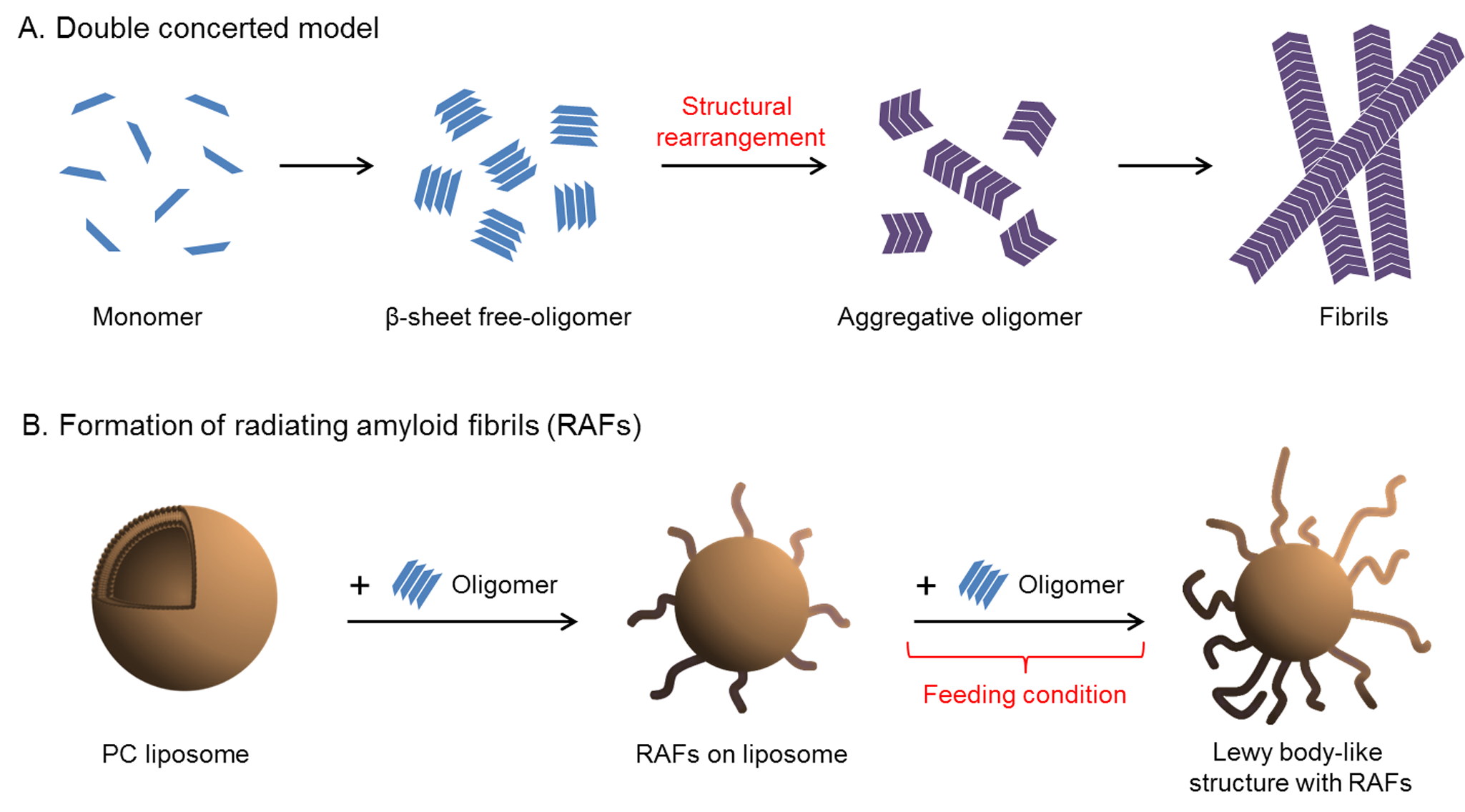

Supplement: Figure S5 — Illustrative scheme of the RAF formation on the surface of liposomes. (A) Double-concerted model explaining the amyloid fibril formation via unit-assembly process of the oligomeric α-synuclein. Soluble monomeric α-synuclein aggregates and forms the β-sheet free oligomers. As these pseudo-stable oligomers sense environmental factors including shear force, organic solvent, and the hydrophilic-hydrophobic interface of lipid membranes, they become more aggregative oligomers with concomitant internal structure rearrangement, which results in the accelerated amyloid fibril formation. (B) Formation of radiating amyloid fibrils (RAFs) on the surface of liposomes. The β-Sheet free oligomers would experience the structural rearrangement at the interface of lipid membranes, and they subsequently form the RAFs on the surface of liposomes. The RAFs are further elongated to form the Lewy-body like (LBL) structure as they are incubated with the oligomeric α-synuclein. (TIF) [file pone.0047580.s005.tif]
